# Comprehensive in vitro analysis evaluating the variable drug–drug interaction risk of rifampicin compared to rifabutin

**DOI:** 10.1007/s00204-023-03531-2

**Published:** 2023-06-07

**Authors:** Julie Nilles, Johanna Weiss, Max Sauter, Walter E. Haefeli, Stephanie Ruez, Dirk Theile

**Affiliations:** 1 Department of Clinical Pharmacology and Pharmacoepidemiology, Heidelberg University Hospital, Im Neuenheimer Feld 410, 69120 Heidelberg, Germany; 2 Boehringer Ingelheim Pharma GmbH & Co. KG, Birkendorfer Str. 65, 88397 Biberach an der Riss, Germany

**Keywords:** Rifampicin, Rifabutin, CYP3A4, ABCB1, Pgp, Induction

## Abstract

Compared to rifampicin (600 mg/day), standard doses of rifabutin (300 mg/day) have a lower risk of drug–drug interactions due to induction of cytochrome P450 3A4 (CYP3A4) or P-glycoprotein (Pgp/*ABCB1*) mediated by the pregnane X receptor (PXR). However, clinical comparisons with equal rifamycin doses or in vitro experiments respecting actual intracellular concentrations are lacking. Thus, the genuine pharmacological differences and the potential molecular mechanisms of the discordant perpetrator effects are unknown. Consequently, the cellular uptake kinetics (mass spectrometry), PXR activation (luciferase reporter gene assays), and impact on CYP3A4 and Pgp/*ABCB1* expression and activity (polymerase chain reaction, enzymatic assays, flow cytometry) were evaluated in LS180 cells after treatment with different rifampicin or rifabutin concentrations for variable exposure times and eventually normalized to actual intracellular concentrations. In addition, inhibitory effects on CYP3A4 and Pgp activities were investigated. While rifampicin is poorly taken up by LS180 cells, it strongly activates PXR and leads to enhanced expression and activity of CYP3A4 and Pgp. In contrast, rifabutin is a significantly less potent and less efficient PXR activator and gene inducer, despite sixfold to eightfold higher intracellular accumulation. Finally, rifabutin is a potent inhibitor of Pgp (IC_50_ = 0.3 µM) compared to rifampicin (IC_50_ = 12.9 µM). Together, rifampicin and rifabutin significantly differ by their effects on the regulation and function of CYP3A4 and Pgp, even when controlled for intracellular concentrations. Rifabutin’s concurrent Pgp inhibitory action might partly compensate the inducing effects, explaining its weaker clinical perpetrator characteristics.

**Supplementary Information:**

The online version contains supplementary material available at 10.1007/s00204-023-03531-2.

## Introduction

Rifampicin and rifabutin are important antibiotics, mostly used to treat mycobacterial infections. Problematic is their ability to reduce the bioavailability of other drugs within a few days of therapy by inducing important drug-metabolizing enzymes (e.g., cytochrome P450 3A4, CYP3A4; Gundert-Remy et al. 2014; Paine et al. 2006) and barrier-forming transporters (e.g., P-glycoprotein, Pgp encoded by *ABCB1*; Lin 2003). For instance, the exposure to other anti-infective drugs such as indinavir (Avihingsanon et al. 2012) or doravirine (Yee et al. 2017) is lowered by approx. 90% after repetitive administration of the 600 mg standard dose of rifampicin. The mechanism of the interaction is seen in the activation of the pregnane X receptor (PXR encoded by *NR1I2*), a nuclear receptor responsible for the expression of these target genes (Prakash et al. 2015; Chen & Raymond 2006). Despite considerable structural similarities and comparable antibiotic activity, rifampicin and rifabutin strikingly differ in their drug–drug interaction potential (Finch et al. 2002; Baciewicz et al. 2008). After repeated administration of the standard dose of 300 mg rifabutin, the bioavailability of co-administered drugs was only slightly reduced compared to rifampicin (indinavir: 34%, Kraft et al. 2004; doravirine: 50%, Khalilieh et al. 2018), indicating significantly weaker inducing properties. Consequently, the interaction potential under rifabutin administration and thus the required dose changes of concomitant drugs are often much lower than under rifampicin treatment.

However, the clinical, cellular, or molecular reasons for these discrepancies are poorly understood. However, to date, there are no data from clinical head-to-head comparisons evaluating the dose-dependent or plasma concentration-dependent induction of CYP3A4 or Pgp activity by rifampicin vs. rifabutin, evaluated with selective substrates of CYP3A (e.g., midazolam) or Pgp (e.g., digoxin, dabigatran etexilate). Several experimental studies have compared the inducing effects of rifampicin and rifabutin (Li et al. 1997; Williamson et al. 2013), but variable drug uptake and unequal intracellular concentrations have mostly been neglected, with only one exception (Dyavar et al. 2020).

Consequently, in this in vitro analysis, rifampicin was compared for the first time with rifabutin following the overall induction pathway: first, the cellular uptake kinetics was investigated by developing a UPLC–MS/MS method to determine the actual intracellular concentrations of the rifamycins. Second, PXR activation dynamics was determined by dual reporter gene assays. Third, *CYP3A4* and Pgp/*ABCB1* mRNA expression was assessed by quantitative real-time polymerase chain reactions. Forth, protein activities (metabolic activity, efflux activity) and potential inhibitory effects were investigated to uncover possible mechanisms counteracting induction. Finally, the results were normalized to actual intracellular concentrations to allow a true comparison of the inducing effects of rifampicin and rifabutin.

## Materials and methods

### Materials

Dulbecco’s Modified Eagle’s Medium (DMEM), RPMI 1640 and fetal calf serum (FCS) were purchased from PAN-Biotech (Aidenbach, Germany). Phosphate-buffered saline (PBS), medium supplements (glutamine, non-essential amino acids, penicillin/streptomycin), ketoconazole, LY335979 (zosuquidar), beta-mercaptoethanol, NH_4_OH and the Gene Elute Mammalian Total RNA Miniprep Kit were purchased from Sigma-Aldrich (Taufkirchen, Germany). Rifampicin, dimethylsulfoxide (DMSO), and crystal violet were purchased from Applichem (Darmstadt, Germany). Rifabutin, 25-*O*-deacetylrifampicin, 25-*O*-deacetylrifabutin, and metformin were supplied by Toronto Research Chemicals (North York, Canada). Doxorubicin was obtained from Biotrend (Köln, Germany). Rhodamine123 was obtained from CalBiochem (Darmstadt, Germany). Calcein-acetoxymethyl ester was obtained from Invitrogen (Karlsruhe, Germany). The Dual-Glo Luciferase Assay System, the pGL4.21 vector, the pGL4.74 [hRluc/TK] *Renilla* vector, the FuGene® HD Transfection reagent, P450-Glo™ CYP Screening Systems with Luciferin-IPA were purchased from Promega Corporation (Madison, WI, USA). The *NR1I2* (NM_003889) human cDNA TrueClone® (pCMV6-XL4 vector, containing the cDNA of the PXR gene *NR1I2*) was obtained from OriGene (Rockville, MD, USA). The RevertAid™ H Minus First Strand cDNA Synthesis Kit was purchased from Thermo Fisher Scientific (Waltham, MA, USA). The Absolute QPCR SYBR Green Mix was supplied by Abgene (Hamburg, Germany). Primers were synthesized by Eurofins MWG Operon (Ebersberg, Germany). Absorbance and luminescence were detected with the SpectraMax iD3 from Molecular Devices (Wokingham, UK). Cell culture flasks and white 96-well plates with white bottom (well-suited for luminescence measurements) were obtained from Greiner (Frickenhausen, Germany). The internal standards ^2^H_8_ rifampicin and ^2^H_6_ rifabutin were purchased from AlsaChim (Strasbourg, France). An Arium® Mini (Sartorius, Göttingen, Germany) ultrapure water system was used to produce purified water. The remaining reagents and solvents, methanol (MeOH), acetonitrile (ACN), and formic acid (FA) were purchased from Biosolve (Valkenswaard, the Netherlands) in the highest available purity.

### Stock solutions

Rifampicin and rifabutin (100 mM stock solutions), ketoconazole, metformin, and zosuquidar (10 mM stock solutions), as well as rhodamine123 (500 µM stock solution) were dissolved in DMSO and stored at − 20 °C. The stock solutions were freshly diluted with medium used in the corresponding experiments. The DMSO concentrations in the assays did not exceed 0.1%.

### Cell lines and culture conditions

#### LS180 cells

For induction experiments, LS180 cells, a human colon adenocarcinoma cell line (available at ATCC, Manassas, VA, USA) was used. This cell line is a well-established model for PXR-mediated induction of genes involved in the metabolism of xenobiotics (Harmsen et al. 2008; Gupta et al. 2008; Weiss et al. 2013; Harper et al. 1991). Thus, its identity was not further verified. Cells were cultured under standard conditions with DMEM, supplemented with 10% FCS, 2 mM glutamine, 100 U/mL penicillin, 100 µg/mL streptomycin sulfate, and 0.1 mM non-essential amino acids.

#### P388 and P388/dx cells

To investigate the potential Pgp inhibition by the rifamycins, the murine monocytic leukaemia cell line P388/dx (overexpressing the murine Pgp counterpart mdr1a/b (Boesch et al. 1991)) and the corresponding parental cell line P388 were used (kindly provided by Dr. D. Ballinari; Pharmacia & Upjohn, Milan, Italy). Cells were cultured under standard conditions with RPMI 1640 cell culture medium supplemented with FCS 10%, 2 mM glutamine, 500 mM β-mercaptoethanol, 100 U/mL penicillin, and 100 µg/mL streptomycin sulphate. The culture medium of the P388/dx cell line was additionally supplemented with doxorubicin (final concentration: 0.45 µM) to maintain mdr1a/b expression. One day before the experiments, P388/dx cells were set to doxorubicin-free medium.

### Growth inhibition assays

Antiproliferative effects of the rifamycins were investigated for all studied incubation periods using proliferation inhibition assays with crystal violet staining as described earlier (Nilles et al. 2022a, b; Peters et al. 2006). Experiments were performed in three independent biological replicates with *n* = 8 wells for each concentration. In all subsequent investigations, only concentrations below the IC_20_ value were used. Consequently, the administered drug concentrations of rifampicin and rifabutin varied between 0.01 and 100 µM in some experiments.

### Quantification of rifamycins in LS180 cells by UPLC–MS/MS

#### UPLC–MS/MS method

To determine the concentration of rifamycins and their major metabolites in LS180 cell homogenates, a UPLC–MS/MS method was developed that simultaneously quantifies rifampicin, rifabutin, 25-*O*-deacetylrifampicin, and 25-*O*-deacetylrifabutin and was validated in compliance with the applicable sections of the EMA (European Medicines Agency, 2009) and FDA (Food and Drug Administration, 2018) recommendations for bioanalytical method validation (validation results provided in Supplemental Material, Tables S1–4). The isotopologues ^2^H_8_-rifampicin and ^2^H_6_-rifabutin were used as internal standards.

The UPLC–MS/MS system consisted of a triple stage quadrupole mass spectrometer (Waters Xevo TQ-S, Milford, MA, USA) and an Acquity Classic UPLC® (Waters). Mass spectrometric analysis was performed by selective reaction monitoring using positive electrospray ionization. The monitored mass transitions were *m/*z 823.12 → 791.2 for rifampicin, *m/*z 831.19 → 799.26 for ^2^H_8_-rifampicin, *m/*z 847.27 → 815.27 for rifabutin, *m/*z 853.33 → 821.27 for ^2^H_6_-rifabutin, and *m/*z 805.4 → 773.33 for 25-*O*-deacetylrifabutin. For 25-*O*-deacteylrifampicin, the mass transition *m/*z 749.34 → 399.17 was monitored, which constitutes the use of a product ion generated during electrospray ionization as precursor. Because of their considerable molecular size, rifamycins were chromatographically separated on a Waters Peptide BEH C18 Column (300 Å, 1.7 µm, 2.1 mm × 50 mm) to foster optimal mass transfer kinetics. The mobile phase was composed of H_2_0/ACN (95/5, *v/v*) + 0.1% FA (eluent A) and ACN + 0.1% FA (eluent B). The separation gradient started with initial conditions of 80% A/20% B and changed after 3 min to 50% A/50% B. The eluent composition was adjusted after 3.1 min to 5% A/95% B and kept for 0.6 min to clean the system before returning to initial conditions over 0.3 min. The total run time was 4 min, and the column was heated to 60 °C. The injection volume was set to 20 µL with a flow rate of 0.5 mL/min.

Two separate weighings each for rifampicin, rifabutin, 25-*O*-deacetylrifampicin, and 25-*O*-deacetylrifabutin were performed and the compounds were dissolved in ACN/H_2_0 (50/50, *v/v*) to prepare calibration standards and quality control (QC) samples. Calibration standard spike solutions were prepared at concentrations of 0.5, 1.5, 5, 15, 50, 150, and 500 ng/mL, which corresponds to sample concentrations of 0.1, 0.3, 1, 3, 10, 30, and 100 ng/mL. QC stock solutions were prepared at 0.5 (LLOQ, lower limit of quantification), 1.5 (low QC), 187.5 (mid QC), and 375 ng/mL (high QC), which corresponds to QC sample concentrations of 0.1, 0.3, 37.5, and 75 ng/mL. Internal standard stock solutions were diluted to obtain a concentration of 10 ng/mL for rifampicin and 1 ng/mL for rifabutin.

### Sample preparation

A pellet of untreated LS180 cells (500,000 cells/sample) was used for calibration standards and QC standard. All samples (uptake samples, calibration standards and QC standards) were lysed with 50 µL MeOH/H_2_O (50/50, v/v) + 5% NH_3_ for 10 min on a shaker. Subsequently, proteins were precipitated with 110 µL ACN (uptake samples) or 100 µL ACN (calibration standards and QC standards). All samples were spiked with 10 µL of the previously diluted internal standards, and standard samples were additionally spiked with 10 µL of calibration standard or QC standard. Afterwards, all samples were centrifuged for 5 min at the highest possible speed (14,000*g*) to remove cell debris from the lysate. One hundred µL of supernatant was transferred to the measurement plate for detection, and the organic phase was evaporated. For measurement, the residue was taken up in 100 µL H_2_O/ACN (80/20, *v/v*) + 0.1% FA. For samples outside the calibration range only 25 µL of supernatant was used and the residue was taken up in 100 µL H_2_O/ACN (80/20, *v/v*) + 0.1% FA. For evaluation, obtained values were multiplied by 4.

### Evaluation of drug uptake kinetics

For the uptake experiments, 20,000 LS180 cells per 96-well were seeded and allowed to attach overnight. On the following day, cells were treated for 6, 24, and 48 h with 0.1, 0.5, 1, 5, or 10 µM rifampicin or rifabutin. After treatment, the medium was removed, the cells were washed twice with ice-cold PBS, and samples were prepared as described before. To determine intracellular rifamycin concentrations, measured compound concentrations were normalized to the total pellet volume. This was achieved by multiplying the measured mean cellular volume of a LS180 cell (CASY cell counter, Schärfe System, Reutlingen, Germany) by the mean cell number of four wells of an identically prepared and treated cell culture plate. Experiments were conducted in duplicate (one plate for uptake experiments and a second plate for normalization via crystal violet staining (Nilles et al. 2022a, b)) with four biological replicates for each concentration.

### PXR activity assay

PXR activity was measured with a reporter gene assay as described previously (Nilles et al. 2022a, b; Weiss et al. 2013). Fifty thousand LS180 cells per well were seeded into white 96-well plates. The next day, each well was transfected with 20 ng of the PXR expression vector (pCMV6-XL4 vector encoding the cDNA of human PXR), 80 ng of the reporter vector (PXR response elements of the CYP3A4 gene, cloned upstream of the open reading frame of firefly luciferase), and 10 ng of the *Renilla* vector (pGL4.74 [hRluc/TK]), which was used for signal normalization (Nilles et al. 2022a, b). Twenty-four hours after transfection, cells were treated with rifampicin or rifabutin for 6, 16, 24, 72, 96, or 120 h at 37 °C. After treatment, luminescence was detected using the Dual-Glo Luciferase assay system according to manufacturer’s instructions with minor changes to the original protocol. Briefly, remaining medium was removed and replaced by 40 µL drug-free medium. Afterwards, 40 µL of firefly detection reagent (luciferin-containing lysis buffer) was added and the cells were incubated for 15 min at room temperature. After incubation, firefly luminescence was detected using a luminometer. To record *Renilla* luminescence, 40 µL of the Stop&Glo reagent (comprising the *Renilla* substrate coelenterazine) was added and the plate was also incubated for 15 min at room temperature.

The net PXR activity was determined by dividing firefly luminescence signals by *Renilla* luminescence signals and normalized to the mean value of the untreated control cells. Experiments were performed in three independent biological replicates with *n* = 4 wells for each concentration.

### Induction of *CYP3A4 *and *ABCB1* and quantification of mRNA expression

After exposure of LS180 cells to variable drug concentrations for 24, 96, or 144 h, total RNA was isolated using the GeneElute Mammalian Total RNA Miniprep Kit according to the manufacturer’s instructions. cDNA was synthesized from total RNA using the RevertAid™ H Minus First Strand cDNA Synthesis Kit (reverse transcriptase-based). A random hexamer primer was used according to the manufacturer’s instructions. Expression levels of *CYP3A4* and *ABCB1* were quantified by real-time reverse transcription (RT) polymerase chain reaction (PCR) with a LightCycler® 480 (Roche Applied Science, Mannheim, Germany) (Albermann et al. 2005; Weiss et al. 2011). PCR conditions and the primer sequences were used as described previously (Albermann et al. 2005; König et al. 2010). Among a set of six housekeeping genes tested, G6PDH (glucose-6-phosphate-dehydrogenase) was the most stable gene (tested with geNorm, version 3.4, Center for Medical Genetics, Ghent, Belgium) and was consequently used for normalization. Data were analyzed as described previously (Albermann et al. 2005). Experiments were performed in four independent biological replicates with technical duplicates (PCR runs) for each concentration.

### Assessment of CYP3A4 activity in LS180 cells

To determine CYP3A4 activity in LS180 cells after rifamycin treatment, 20,000 cells per well were seeded into 96-well plates and allowed to grow for 3 days until 70% confluency. Subsequently, cells were treated with rifampicin or rifabutin, with different concentrations for 24, 72, or 96 h. For the detection of CPY3A4 activity, the P450-Glo™ CYP3A4 assay system (Promega Corporation, Madison, WI, USA) was used according to the manufacturer's instructions. Cells were washed twice with PBS and then pre-incubated with PBS + 2% FCS for 30 min at 37 °C. PBS was removed and replaced by 50 µL of 3 µM luciferin-IPA (selective CYP3A4 substrate) and incubated for 60 min at 37 °C. After incubation, 25 µL supernatant from each well was transferred to a white 96-well plate and 25 µL detection reagent (containing luciferase) was added. Cells were subsequently incubated for 20 min at room temperature. Luminescence was recorded immediately and normalized to cell number using crystal violet staining (Theile et al. 2021). CYP3A4 activity was normalized to untreated controls. Experiments were performed in three to four independent biological replicates with three (72, 96 h) or four (24 h) technical replicates each.

### Evaluation of intracellular concentration-normalized effects

To control for variable drug uptake or accumulation, biological effects (PXR activity, *CYP3A4* and *ABCB1* mRNA expression and CYP3A4 activity) of 24 h drug exposure were normalized to intracellular concentrations, using the linear relationship obtained from the 24 h uptake experiments.

### Assessment of Pgp activity in LS180 cells

To assess the induction of Pgp activity, 200,000 LS180 cells per well were seeded in a 6-well plate, cultured for 3 days and subsequently exposed to rifamycins for 144 h. After treatment, cells were harvested and subjected to the rhodamine efflux assay as described previously (Theile et al. 2013; Weiss et al. 2008). Briefly, cells were incubated with the fluorescent Pgp substrate rhodamine123 (0.4 µM) on a rotary shaker for 30 min at 37 °C under protection from light. Subsequently, cells were incubated with the selective Pgp inhibitor zosuquidar (10 µM) for 50 min at 37 °C on a rotary shaker. Medium without zosuquidar was used as a negative control. All washing steps were performed with ice-cold PBS at 4 °C. Intracellular rhodamine123 fluorescence was determined using a flow cytometer (MACSQuant analyzer 10, Miltenyi Biotec, Bergisch Gladbach, Germany). Thirty thousand cells were used for the analysis. For data evaluation, the ratio of zosuquidar-inhibited samples to non-inhibited samples of the rifamycin experiment was calculated and normalized to the ratio of untreated cell controls (no rifamycin treatment). Experiments were performed in three independent biological replicates for each concentration.

### CYP3A4 inhibition assay

Because concurrent CYP3A4 inhibition could weaken the net effect of induction, CYP3A4 inhibition by rifampicin or rifabutin was evaluated using the P450-Glo™ CYP3A4 Assay and Screening System provided by Promega Corporation (Madison, WI, USA) according to the manufacturer’s instructions. For this purpose, rifamycins were tested between 0.5 to 50 µM. Ketoconazole (10 µM, strong CYP3A4 inhibitor; Weiss et al. 2022) was used as a positive control. A reaction mixture without CYP3A4 was incubated as negative control. Luminescence signals were normalized to the untreated cell control. Experiments were performed in three independent biological replicates with *n* = 3 for each concentration.

### Pgp inhibition assay

Because concurrent Pgp inhibition could counteract the inducing effects observed in vivo, Pgp inhibitory properties of rifampicin and rifabutin were additionally assessed in P388/dx cells (overexpressing the murine Pgp counterpart called mdr1a/b) and corresponding parental P388 cells. Pgp activity was determined using a calcein assay as described previously (Fröhlich et al. 2004) with zosuquidar (10 µM) as a positive control. Fluorescence signals were normalized to the untreated cell control. Experiments were performed in three independent biological replicates with *n* = 8 for each concentration.

### Statistics

Time-dependent PXR activation, *CYP3A4* and *ABCB1* mRNA expression, and CYP3A4 and Pgp activity was evaluated by ANOVA with non-parametric Kruskal–Wallis test using GraphPad Prism version 9.1 (GraphPad Software Inc., La Jolla, CA, USA). Comparisons of rifampicin or rifabutin treatments were evaluated by Student’s *t* test using GraphPad Prism. Data were plotted with GraphPad Prism according to a hyperbolic (x is concentration) model for uptake kinetics and a sigmoidal *E*_max_ model (four parameter-logistic equation; variable slope) for induction experiments. A *P* value < 0.05 was considered significant.

## Results

### Uptake and metabolism of rifamycins in LS180 cells

**Fig. 1:**
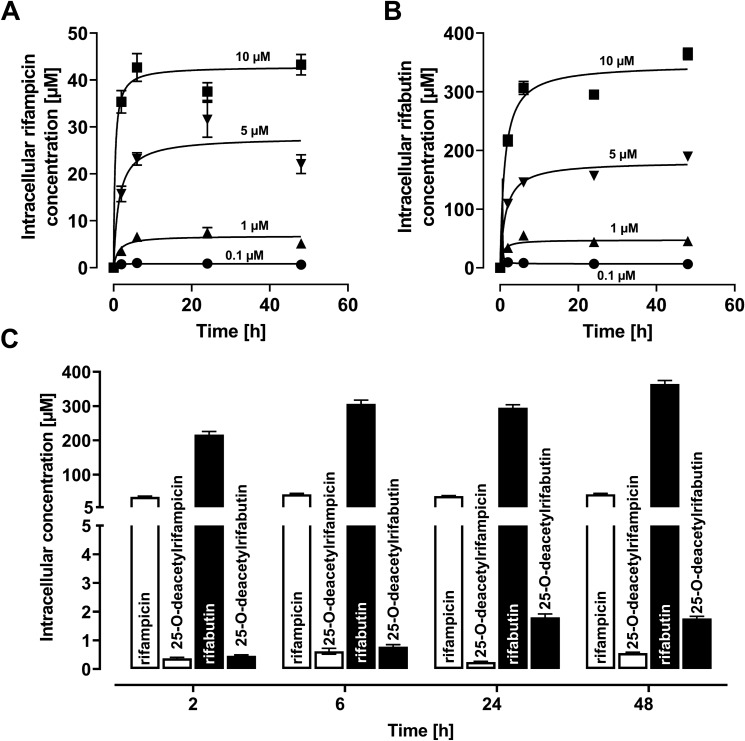
Intracellular concentrations of rifampicin (**A**) or rifabutin (**B**) in LS180 cells after drug exposure for 2, 6, 24, or 48 h (0.1 µM = circles; 1 µM = triangles, up; 5 µM = triangles, down; 10 µM = squares). Data points were fitted according to a hyperbola model. **C** Intracellular concentrations of rifampicin and its metabolite 25-*O*-deacetylrifampicin (light bars) or rifabutin and its metabolite 25-*O*-deacetylrifabutin (dark bars) after exposure to 10 µM parent drug for 2, 6, 24, or 48 h. Data shown are the mean ± SEM of four independent biological replicates

Intracellular drug concentrations in LS180 cells increased in a concentration-dependent manner (Fig. 1), reaching a concentration equilibrium after 2 h for rifampicin and after 6 h for rifabutin. Rifabutin accumulated 6.1-fold (2 h), 7.2-fold (6 h), 7.9-fold (24 h), and 8.4-fold (48 h) higher than rifampicin (*P* < 0.0001 for all uptake times). Concurrently, only minimal generation of the main deacetyl metabolites was recorded (Fig. 1C); the molar metabolite-to-parent ratio was 0.01 for rifampicin and 0.002 to 0.006 for rifabutin, so drug metabolism (and a possible influence of metabolites on the results) was considered negligible in this experiment.

### PXR activity in LS180 cells

**Fig. 2:**
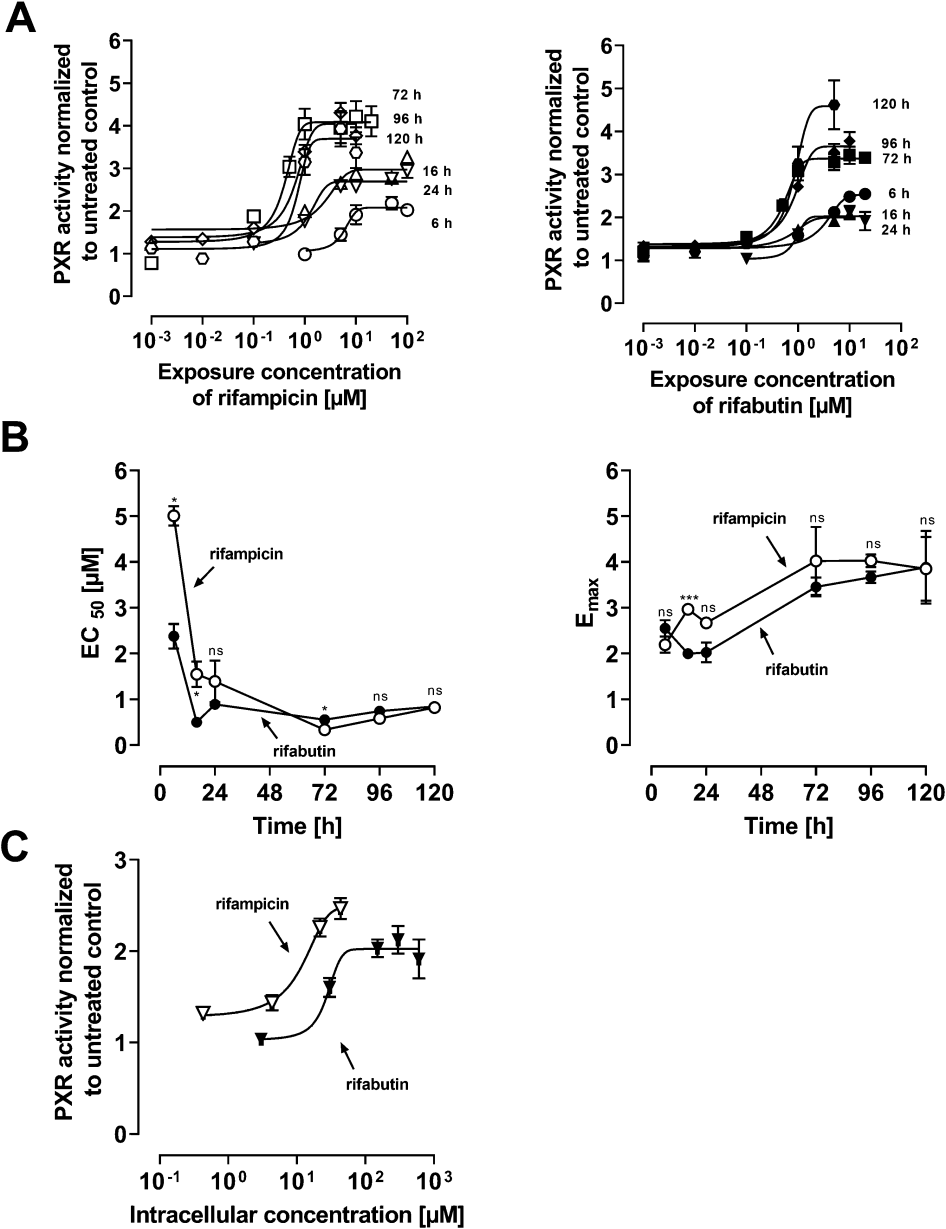
Relative pregnane × receptor (PXR) reporter activity in LS180 cells after rifamycin treatment. Data shown are the mean ± SEM of three independent biological replicates with *n* = 4 replicates for each concentration. EC_50_ and *E*_max_ values were calculated according to an *E*_max_ model (four parameter-logistic equation; variable slope). **A** PXR activation after 6, 16, 24, 72, 96, or 120 h exposure to rifampicin (left) or rifabutin (right). **B** EC_50_ data (left) and *E*_max_ data (right) of PXR activation for each timepoint after rifampicin (open circles) or rifabutin (filled circles) treatment. Data points are simply connected. Significant differences in EC_50_ and *E*_max_ between the drugs at the respective timepoint were evaluated by Student’s *t* test using GraphPad Prism version 9.1; *P < 0.05, ***P < 0.001, ns = not significant. **C** Relative PXR activity in LS180 cells after intracellular exposure to rifampicin (open circles) or rifabutin (closed circles) for 24 h

After treatment with rifamycins, PXR activity in LS180 cells increased as a function of exposure concentration (Fig. 2A). The EC_50_ (potency) and *E*_max_ (efficacy) values for both drugs reached their approximate maximum after 72 h without changing further in the following 48 h (Fig. 2B). Comparing both substances, differences in potency and efficacy were primarily detectable after short incubation times (6–72 h). Normalizing the 24 h exposure effects to actual intracellular concentrations (Fig. 2C) showed that rifampicin is a more potent (EC_50_: *P* = 0.004) but equally effective (*E*_max_: *P* > 0.05) PXR activator as rifabutin.

### CYP3A4 and ABCB1 mRNA expression in LS180 cells

**Fig. 3:**
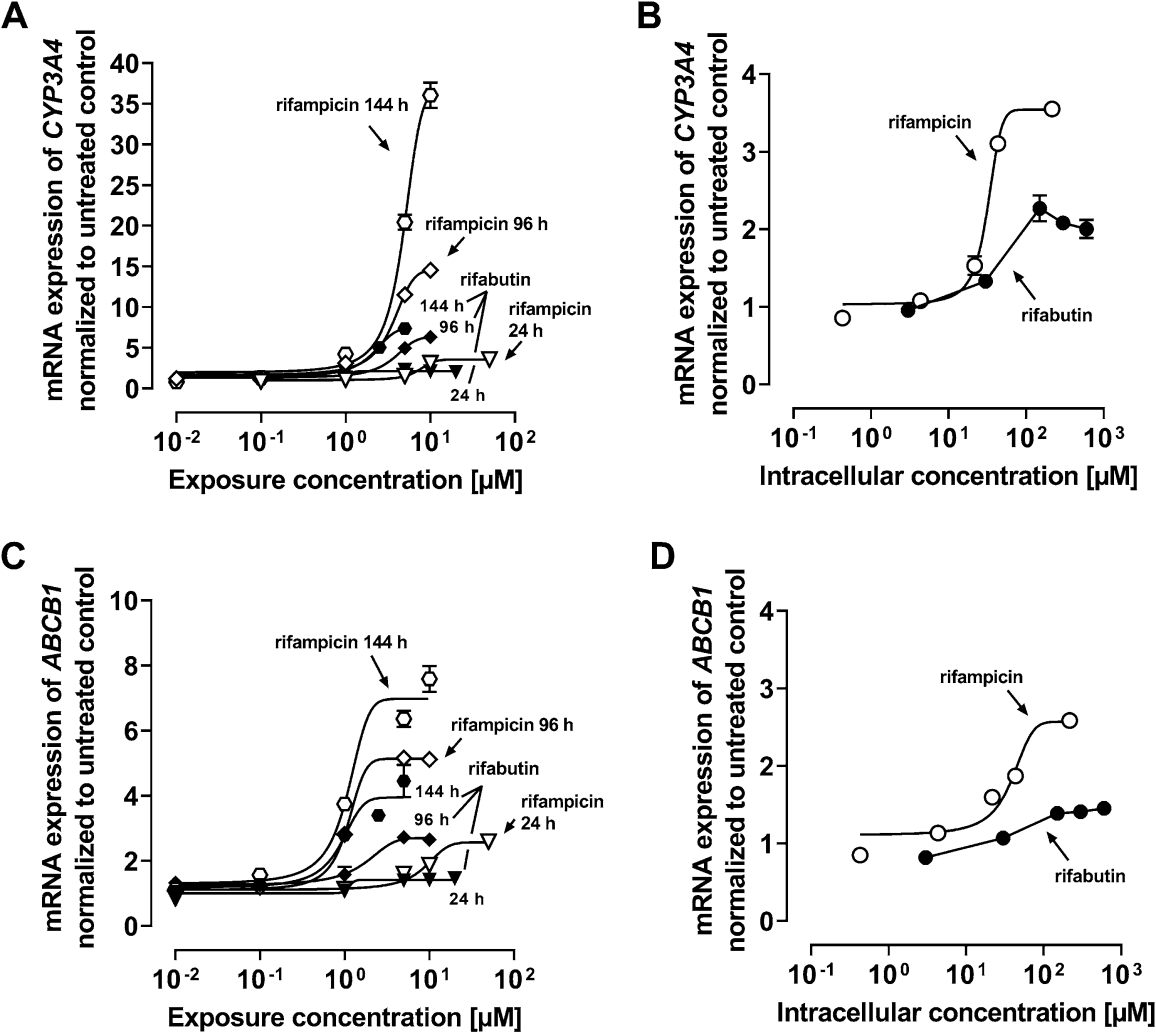
mRNA expression of *CYP3A4* (**A**) or *ABCB1* (**C**) in LS180 cells after exposure to rifampicin (open symbols) or rifabutin (filled symbols) for 24, 96, and 144 h. mRNA expression of *CYP3A4* (**B**) and *ABCB1* (**D**) in LS180 cells after exposure to rifampicin (open symbols) or rifabutin (filled symbols) for 24 h, normalized to intracellular concentrations. Data shown are the mean ± SEM of four independent biological replicates. Data were fitted according to an *E*_max_ model (four parameter-logistic equation; variable slope). Data points for rifabutin in part (**B**) and (**D**) were simply connected, because fitting was not possible. Individual EC_50_ and *E*_max_ values are listed in Supplemental Material Table S5

Both rifamycins induced *CYP3A4* and *ABCB1* with increasing extracellular concentrations (Fig. 3A: *CYP3A4*; C: *ABCB1*, Supplemental Material Table S5). With longer exposure times, there was no increase in *CYP3A4* induction potency (decrease of EC_50_), but a significant increase in induction efficacy (*E*_max_, *P* < 0.01). In contrast, gene-inducing potencies and efficacies for *ABCB1* increased with longer exposure (24 h vs. 144 h, *P* < 0.05). Importantly, differences in *CYP3A4* and *ABCB1* induction were particularly obvious when mRNA levels were related to the corresponding intracellular drug concentrations after 24 h exposure: Rifampicin potently (EC_50_ of 32.4 ± 2.0 µM) increased *CYP3A4* mRNA levels with an *E*_max_ of 3.5-fold (± 0.1) compared to untreated control, whereas *ABCB1* was induced with an EC_50_ of 37.4 µM (± 2.0) and *E*_max_ of 2.6-fold (± 0.1). In contrast, the corresponding parameters could not be computed for rifabutin because of a lacking sigmoidal character. Overall, rifampicin was the more potent *CYP3A4* and *ABCB1* inducer than rifabutin, which is particularly evident when the results are normalized to actual intracellular drug concentrations.

### Metabolic CYP3A4 activity and Pgp efflux function in LS180 cells

**Fig. 4:**
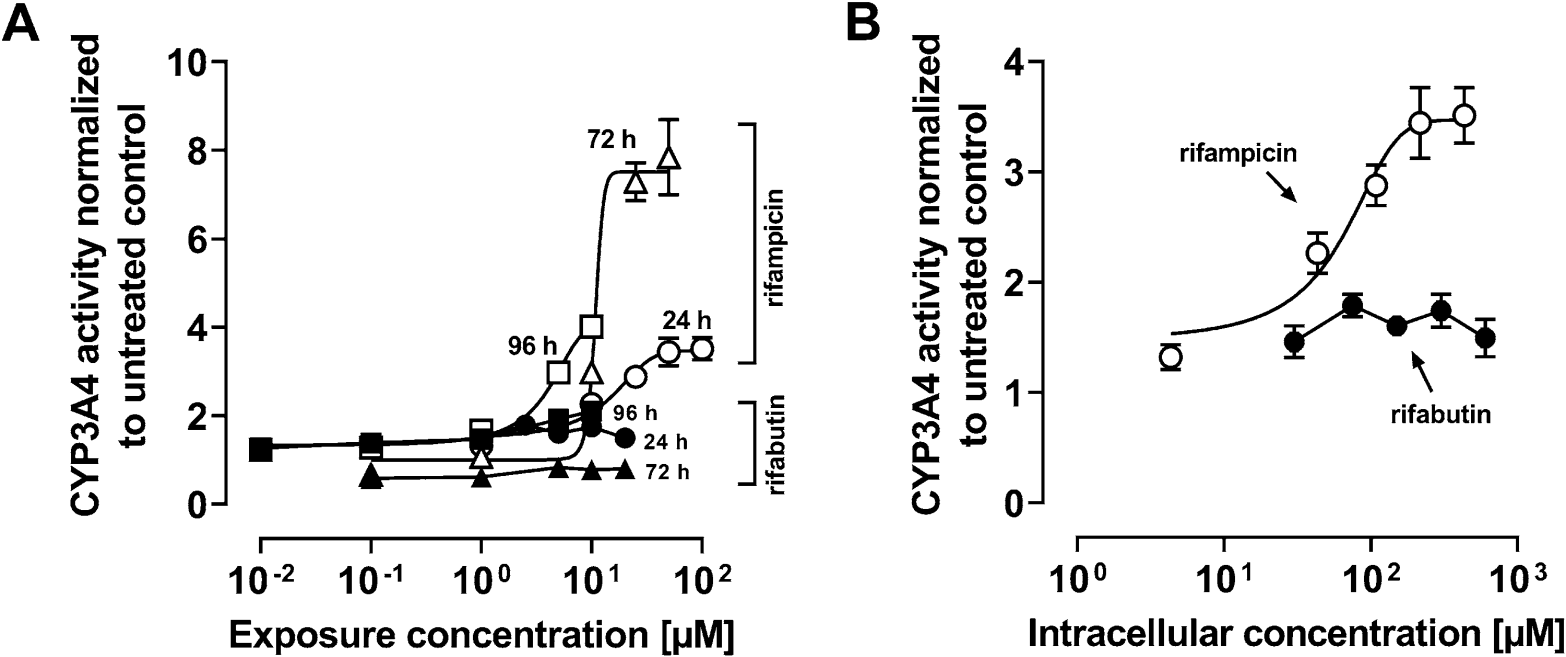
**A** CYP3A4 activities in LS180 cells after exposure to rifampicin (open symbols) or rifabutin (filled symbols) for 24, 72, or 96 h. **B** CYP3A4 activities in LS180 cells after intracellular exposure to rifampicin (open symbols) or rifabutin (filled symbols) for 24 h. Data shown are the mean ± SEM of four independent biological replicates with n = 4 replicates for each concentration. The data were fitted to an *E*_max_ model (four parameter-logistic equation; variable slope); data points for rifabutin in part B were simply connected, because fitting was not possible. Individual EC_50_ and *E*_max_ values are listed in Supplemental Material Table S6

Rifampicin caused an increase in metabolic CYP3A4 activity (Fig. 4A). The exposure time had no impact on rifampicin’s EC_50_, whereas efficacy reached its maximum after 72 h (*E*_max_ 24 h vs. 72 h, *P* = 0.039; 24 h vs. 96 h not significant, Supplemental Material Table S6). In contrast, there was no detectable increase in CYP3A4 activity after exposure to rifabutin at any timepoint (Fig. 4A). Looking at the 24 h data normalized to intracellular concentrations, an EC_50_ value of 42.8 (± 1.5) µM and an *E*_max_ of 3.5-fold (± 0.8) were observed for rifampicin (Fig. 4), while no concentration dependence was observed for rifabutin.

**Fig. 5:**
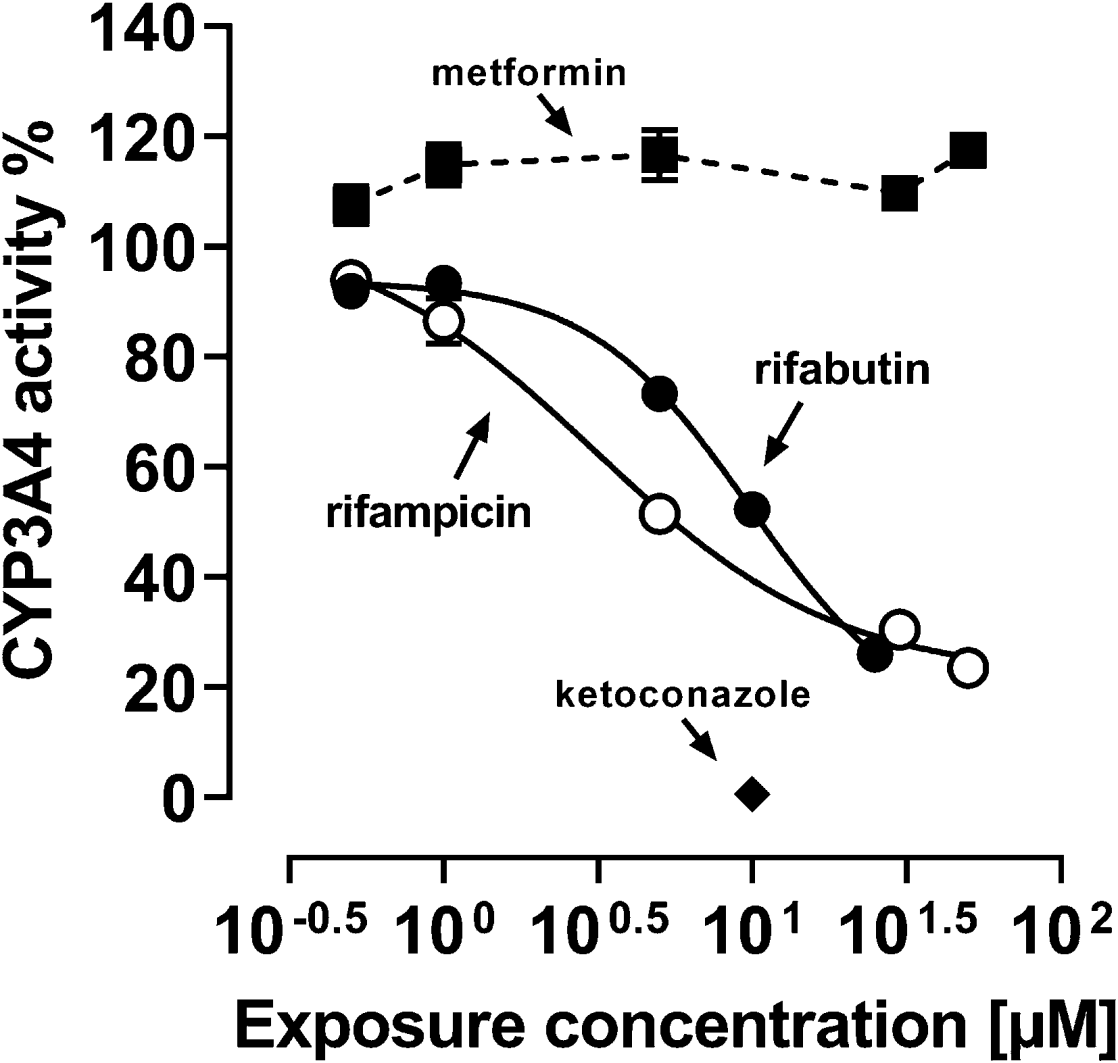
CYP3A4 inhibition by rifampicin (open circles) or rifabutin (filled circles), normalized to untreated control. Metformin (filled squares) was used as an inert non-inhibitor control and ketoconazole (10 µM, filled diamond) as a strong CYP3A4 inhibitor positive control. Data shown are the mean ± SEM of 3 independent replicates with *n* = 3. The data were fitted to an *E*_max_ model (four parameter-logistic equation; variable slope). The data points for metformin were simply connected. Individual EC_50_ and *E*_max_ values are listed in

To evaluate whether overlapping CYP3A4 inhibitory effects modulate the recorded net effects, CYP3A4 inhibition was assessed in a separate experimental setup using a commercial microsome-based luminescence assay (Fig. 5). Both drugs lowered CYP3A4 activity by approx. 80%. However, rifampicin was more potent (IC_50_ of rifampicin: 2.9 ± 0.9 µM; rifabutin: 10.6 ± 2.9 µM; *P* = 0.022; Supplemental Material Table S6). The positive control ketoconazole completely abolished CYP3A4 activity (10 µM: 0.6 ± 0.4% activity compared to untreated control) and the negative control metformin had no effect.

**Fig. 6:**
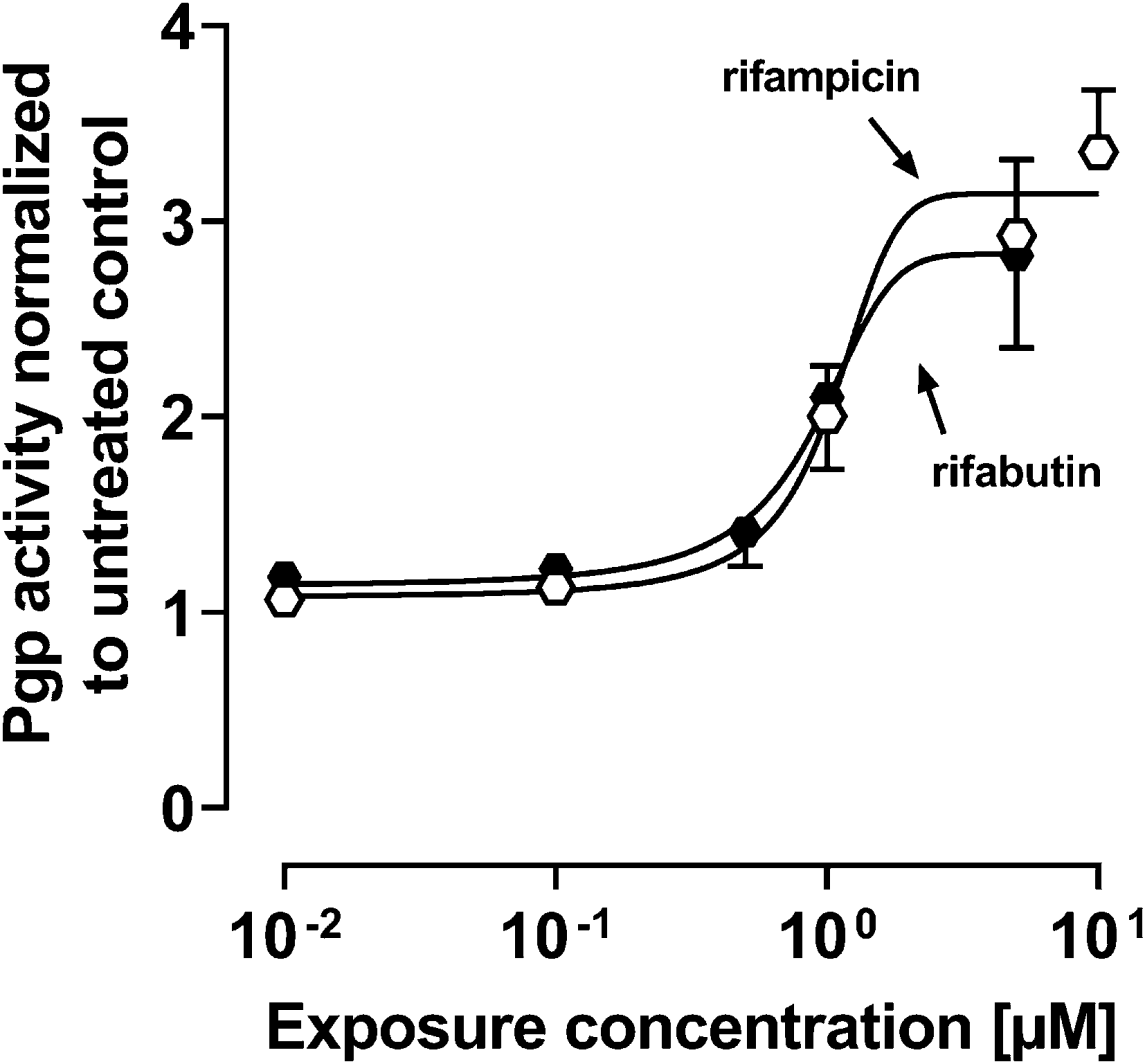
Pgp activity after exposure of LS180 cells to rifampicin (open symbols) or rifabutin (filled symbols) for 144 h. Data shown are the mean ± SEM of three independent biological replicates. The data were fitted to an *E*_max_ model (four parameter-logistic equation; variable slope). Individual EC_50_ and *E*_max_ values are listed in Supplemental Material Table S7

The functional analysis of Pgp activity was determined by rhodamine123 efflux and detection by flow cytometry after 144 h drug exposure. Both drugs increased Pgp activity depending on the exposure concentration (Fig. 6) without significant pharmacodynamic differences (Supplemental Material, Table S7).

**Fig. 7:**
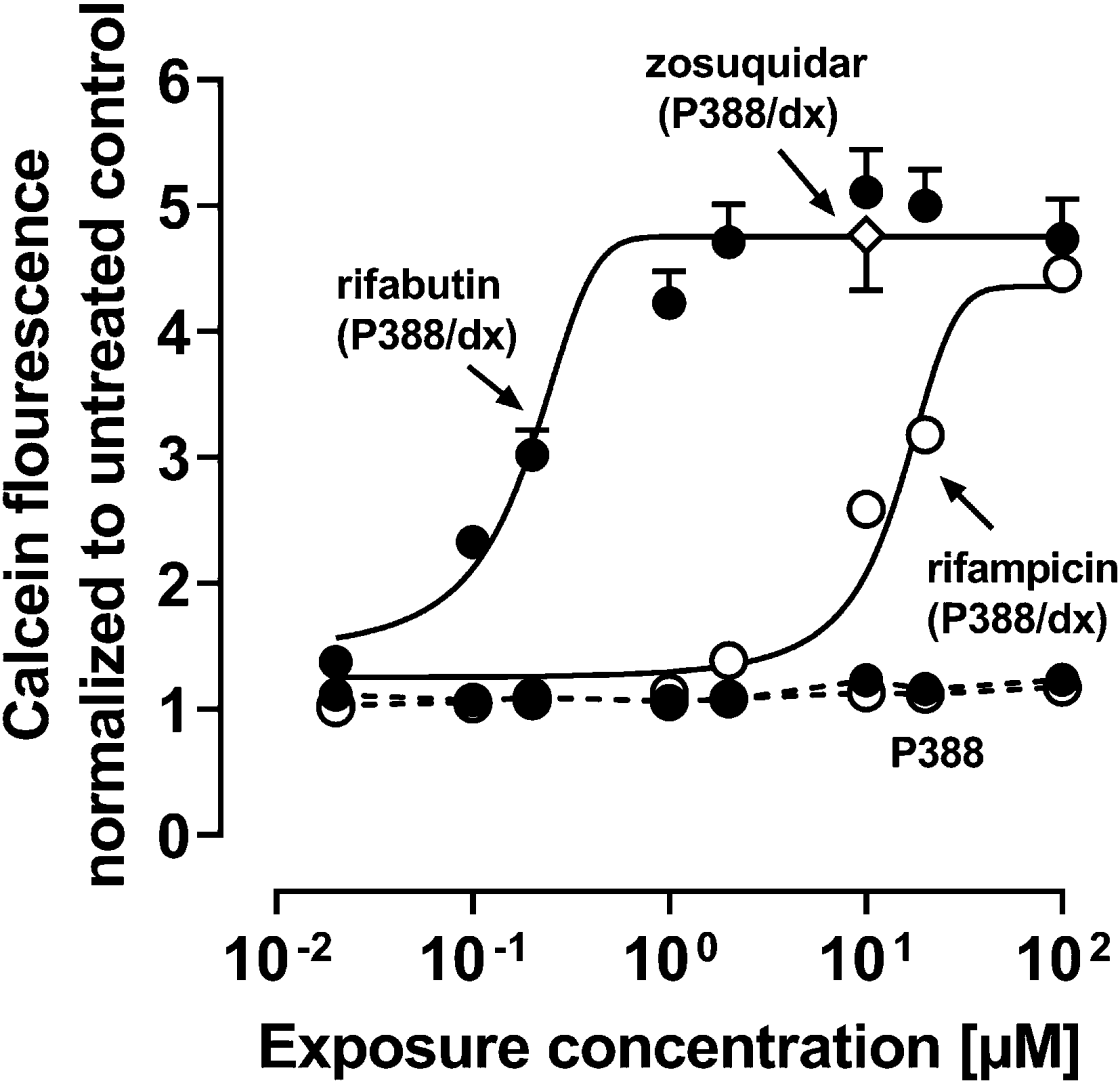
Pgp inhibition by rifampicin (open circles) or rifabutin (filled circles) in P388/dx cells (*E*_max_ model fit, solid line) and P388 cells (simply connected by dashed lines) cells. Zosuquidar (10 µM; white diamond, evaluated in P388/dx cells) was used as a positive control. Data shown are the mean ± SEM of independent biological replicates with *n* = 8. Data were fitted to an *E*_max_ model (four parameter-logistic equation with variable slope). Individual IC_50_ values are listed in Supplemental Material Table S7

To determine the potential inhibition of Pgp by rifampicin or rifabutin, calcein efflux in murine P388 and P388/dx cells was assessed (Supplemented Material Table S7). In P388 cells, intracellular calcein fluorescence remained unchanged (Fig. 7), whereas rifabutin potently inhibited efflux in P388/dx cells (IC_50_: 0.3 ± 0.1 µM) and to a comparable extent as the positive control zosuquidar. Rifampicin inhibited mdr1a/b as well but was substantially less potent than rifabutin (IC_50_ = 12.9 ± 4.1 µM, *P* = 0.013).

## Discussion

The repeated administration of a very low, subtherapeutic dose of 10 mg rifampicin to humans can decrease the area-under-the-curve (AUC) and maximum plasma concentration (*C*_max_) of midazolam (paradigm CYP3A4 substrate) or dabigatran etexilate (paradigm Pgp substrate) to 50% (Lutz et al., CPT 2018a). In contrast, 300 mg rifabutin is needed to alter midazolam kinetics comparably, while dabigatran etexilate is not affected (Lutz et al., CPT 2018b). Accordingly, our study investigated possible underlying reasons for the highly differing induction property of these two antibiotics in vitro. Because induction is mediated by (intracellular) nuclear receptors, different cellular drug uptake could theoretically explain the different induction effects. Remarkably, in our study the accumulation of rifabutin in LS180 cells was sixfold to eightfold higher than that of rifampicin, confirming previous observations (Dyavar et al. 2020) and ruling out the possibility that differences were simply due to unequal cellular drug uptake (Fig. 1). Despite considerably lower intracellular concentrations, rifampicin was the more potent and efficient PXR activator (Fig. 2), causing significantly higher *CYP3A4* and *ABCB1* mRNA levels (Fig. 3). Nevertheless, *CYP3A4* mRNA levels were still increased twofold by rifabutin, confirming the inducing properties of this compound. Surprisingly, rifabutin, however, had only little effect on CYP3A4 activity, whereas rifampicin substantially increased CYP3A4-mediated metabolism. Given this lack of activity enhancement by rifabutin, concurrent enzyme inhibition was hypothesized. Both rifampicin and rifabutin inhibited CYP3A4 comparably (Fig. 5), being in excellent agreement to previous findings on rifampicin (Kajosaari et al. 2005). Consequently, the observed lack of CYP3A4 activity enhancement after rifabutin treatment cannot be explained by strong enzyme inhibition, because both rifamycins exhibited similar inhibitory properties. Furthermore, these properties did not reduce the metabolism of the paradigm CYP3A substrate midazolam in the case of rifampicin (Yoshikado et al. 2017). In vitro, both rifamycins induced Pgp activity comparably (Fig. 6), but the known clinical net effects on the pharmacokinetics of Pgp substrates (e.g., dabigatran etexilate) considerably differ between rifampicin and rifabutin (Lutz et al., Clin Pharmacol Ther 2018a; Lutz et al., Clin Pharmacol Ther 2018b), suggesting a concurrent Pgp inhibition by rifabutin. In our experiments, rifabutin was a strong Pgp inhibitor, exceeding Pgp inhibition by rifampicin by more than an order of magnitude (EC_50_ rifabutin: 0.3 µM; EC_50_ rifampicin: 12.9 µM, Fig. 7). This finding is in line with our recently published experimental data (rifabutin-mediated inhibition of human Pgp, over-expressed in porcine kidney cells) and a physiology-based pharmacokinetic model of clinical data (rifabutin–dolutegravir interaction) (Theile et al. 2023), and underlines the potential clinical relevance given the expected drug concentrations in the gut lumen (600 mg rifampicin: approx. 2.9 mM; 300 mg rifabutin: approx. 1.4 mM; Zhang et al. 2008).

In summary, our series of experiments has shown that the effects of rifampicin at comparable intracellular concentrations differ significantly from those of rifabutin and could in part be due to a potent inhibition of Pgp by rifabutin, whereas it is unlikely caused by the weak inhibition of CYP3A4. However, additional molecular mechanisms could also cause such differences. For instance, the variable recruitment of co-activators involved in rifamycin-mediated PXR activation could play a crucial role. So far, different co-activators have been described that modulate the transcriptional induction; among them are the steroid receptor coactivator-1 (Li and Chiang 2006; Pavek 2016), the peroxisome proliferator-activated receptor gamma coactivator-1α (Li and Chiang 2006; Pavek 2016), and the hepatocyte nuclear factor 4α (Li and Chiang 2006; Tirona et al. 2003; Pavek 2016). Moreover, regulatory micro-RNA species can bind the CYP3A4 mRNA and thus block CYP3A4 protein synthesis or foster mRNA degradation (Li et al. 2019; Pan et al. 2009; Ramamoorthy et al. 2013). Theoretically, rifabutin could be an inducer of such CYP3A4-regulating micro-RNA species, explaining the low CYP3A4 activity during rifabutin induction.

This study has limitations: first, a tumor cell line was used. However, LS180 cells are known to be an excellent standard model for PXR-mediated induction of CYP3A4 and Pgp, being superior to liver cell lines (Huh, HepG2) and an adequate substitute for primary hepatocytes (Harmsen et al. 2008; Gupta et al. 2008; Brandin et al. 2007; Yamasaki et al. 2009). Second, metabolism of the parent compounds in vivo might modulate the net inducing effects and models should reflect this accordingly. In LS180 cells, formation of the main 25-*O*-deacetylated metabolites was very low and in agreement with the findings in ex vivo primary human hepatocyte models (Dyavar et al. 2020). Actually, the lack of drug metabolism is one of this study’s strengths, because it allowed for a targeted analysis of the parent drugs without interfering metabolite effects.

## Conclusion

This study showed that rifampicin and rifabutin differ significantly by their effects on CYP3A4 and Pgp regulation and function at similar intracellular concentrations. Overall, rifampicin exhibited stronger effects than rifabutin at the main levels of induction (PXR activation, *CYP3A4*/*ABCB1* mRNA enhancement, CYP3A4 enzyme activity). Rifabutin stands out by two important in vitro findings: first, it does not enhance CYP3A4 metabolic activity despite elevated *CYP3A4* mRNA levels. Second, rifabutin is a potent inhibitor of Pgp. These findings can probably partly explain the weaker clinical perpetrator characteristics of rifabutin compared to rifampicin.

## Supplementary Information

Below is the link to the electronic supplementary material.

Supplementary file1 (DOCX 28 KB)

## Data Availability

All data supporting the findings of this study are available within the paper and its Supplementary Information.
