# Supplementary material for: Comprehensive in vitro analysis evaluating the variable drug–drug interaction risk of rifampicin compared to rifabutin
Source: Arch Toxicol. 2023 Jun 7;97(8):2219–30. doi: 10.1007/s00204-023-03531-2 (PMC10322781; doi:10.1007/s00204-023-03531-2)
Supplement: Supplementary file 1 — Supplementary file1 (DOCX 28 KB) [file 204_2023_3531_MOESM1_ESM.docx]

**Comprehensive in vitro analysis evaluating the variable drug-drug interaction risk of rifampicin compared to rifabutin**

Julie Nilles^1,2^, Johanna Weiss^1^, Max Sauter^1^, Walter E. Haefeli^1^, Stephanie Ruez^2^, Dirk Theile^1^

1) Department of Clinical Pharmacology and Pharmacoepidemiology, Heidelberg University Hospital, Im Neuenheimer Feld 410, 69120 Heidelberg, Germany

2) Boehringer Ingelheim Pharma GmbH & Co. KG, Birkendorfer Str. 65, 88397 Biberach an der Riss, Germany.

**Supplemental Material**

**Table S1**: Validation results of the UPLC-MS/MS quantification method for rifampicin in LS180 cells.

| **Rifampicin** | | **LLOQ**  0.100 ng/mL | **Low QC**  0.300 ng/mL | **Mid QC**  37.5 ng/mL | **High QC**  75.0 ng/mL |
| --- | --- | --- | --- | --- | --- |
| Within-batch | | | | | |
| 1 | Mean [ng/mL] | 0.113 | 0.303 | 36.5 | 75.3 |
|  | Accuracy % | 113 | 101 | 97.3 | 101 |
|  | Precision % | 8.32 | 14.6 | 1.43 | 0.471 |
| 2 | Mean [ng/mL] | 0.105 | 0.298 | 37.9 | 78.7 |
|  | Accuracy % | 105 | 99.4 | 101 | 105 |
|  | Precision % | 4.76 | 3.58 | 1.09 | 1.96 |
| 3 | Mean [ng/mL] | 0.113 | 0.269 | 39.9 | 80.4 |
|  | Accuracy % | 113 | 89.7 | 106 | 107 |
|  | Precision % | 11.6 | 6.74 | 1.41 | 3.21 |
| Batch-to-batch | | | | | |
|  | Mean [ng/mL] | 0.111 | 0.290 | 38.1 | 78.1 |
|  | Accuracy % | 111 | 96.7 | 102 | 104 |
|  | Precision % | 9.54 | 11.1 | 3.94 | 3.51 |

LLOQ: lower limit of quantification; QC: quality control. Validation followed the applicable sections of the FDA and EMA recommendations. All experiments were performed with n = 6.

**Table S2**: Validation results of the UPLC-MS/MS quantification method for rifabutin in LS180 cells.

| **Rifabutin** | | **LLOQ**  0.100 ng/mL | **Low QC**  0.300 ng/mL | **Mid QC**  37.5 ng/mL | **High QC**  75.0 ng/mL |
| --- | --- | --- | --- | --- | --- |
| Within-batch | | | | | |
| 1 | Mean [ng/mL] | 0.115 | 0.310 | 35.7 | 73.4 |
|  | Accuracy [%] | 115 | 103 | 95.2 | 97.9 |
|  | Precision [%] | 13.0 | 3.72 | 1.19 | 0.632 |
| 2 | Mean [ng/mL] | 0.112 | 0.252 | 34.5 | 69.6 |
|  | Accuracy [%] | 112 | 83.9 | 92.0 | 92.8 |
|  | Precision [%] | 15.9 | 8.08 | 1.50 | 2.94 |
| 3 | Mean [ng/mL] | 0.113 | 0.334 | 33.9 | 66.5 |
|  | Accuracy [%] | 113 | 111 | 90.4 | 88.7 |
|  | Precision [%] | 23.5 | 8.08 | 8.56 | 6.30 |
| Batch-to-batch | | | | | |
|  | Mean [ng/mL] | 0.113 | 0.299 | 35.0 | 67.6 |
|  | Accuracy [%] | 101 | 102 | 93.4 | 90.1 |
|  | Precision [%] | 9.61 | 6.07 | 5.32 | 5.26 |

LLOQ: lower limit of quantification; QC: quality control. Validation followed the applicable sections of the FDA and EMA recommendations. All experiments were performed with n = 6.

**Table S3**: Validation results of the UPLC-MS/MS quantification method for 25-O-deacetylrifampicin in LS180 cells.

| **25-O-deacetylrifampicin** | | **LLOQ**  0.100 ng/mL | **Low QC**  0.300 ng/mL | **Mid QC**  37.5 ng/mL | **High QC**  75.0 ng/mL |
| --- | --- | --- | --- | --- | --- |
| Within-batch | | | | | |
| 1 | Mean [ng/mL] | 0.103 | 0.293 | 38.2 | 79.2 |
|  | Accuracy [%] | 103 | 97.8 | 102 | 106 |
|  | Precision [%] | 7.21 | 1.61 | 4.53 | 2.22 |
| 2 | Mean [ng/mL] | 0.113 | 0.313 | 37.6 | 74.4 |
|  | Accuracy [%] | 113 | 104 | 100 | 99.2 |
|  | Precision [%] | 4.16 | 4.39 | 1.49 | 2.18 |
| 3 | Mean [ng/mL] | 0.0890 | 0.267 | 42.6 | 86.7 |
|  | Accuracy [%] | 89.0 | 89.0 | 114 | 116 |
|  | Precision [%] | 9.48 | 6.11 | 1.70 | 2.92 |
| Batch-to-batch | | | | | |
|  | Mean [ng/mL] | 0.102 | 0.291 | 39.5 | 80.1 |
|  | Accuracy [%] | 102 | 97.1 | 105 | 107 |
|  | Precision [%] | 12.0 | 7.81 | 6.36 | 6.81 |

LLOQ: lower limit of quantification; QC: quality control. Validation followed the applicable sections of the FDA and EMA recommendations. All experiments were performed with n = 6.

**Table S4**: Validation results of the UPLC-MS/MS quantification method for 25-O-deacetylrifabutin in LS180 cells.

| **25-O-deacetylrifabutin** | | **LLOQ**  0.100 ng/mL | **Low QC**  0.300 ng/mL | **Mid QC**  37.5 ng/mL | **High QC**  75.0 ng/mL |
| --- | --- | --- | --- | --- | --- |
| Within-batch | | | | | |
| 1 | Mean [ng/mL] | 0.103 | 0.322 | 35.7 | 70.8 |
|  | Accuracy [%] | 103 | 107 | 95.2 | 94.4 |
|  | Precision [%] | 4.56 | 1.16 | 1.06 | 0.810 |
| 2 | Mean [ng/mL] | 0.110 | 0.302 | 34.1 | 64.9 |
|  | Accuracy [%] | 110 | 101 | 91.0 | 86.5 |
|  | Precision [%] | 5.25 | 6.47 | 1.82 | 2.82 |
| 3 | Mean [ng/mL] | 0.091 | 0.292 | 35.2 | 67.1 |
|  | Accuracy [%] | 90.7 | 97.4 | 93.9 | 89.5 |
|  | Precision [%] | 6.67 | 4.63 | 8.31 | 6.03 |
| Batch-to-batch | | | | | |
|  | Mean [ng/mL] | 0.101 | 0.305 | 35.0 | 67.6 |
|  | Accuracy [%] | 101 | 102 | 93.4 | 90.1 |
|  | Precision [%] | 9.61 | 6.07 | 5.32 | 5.26 |

LLOQ: lower limit of quantification; QC: quality control. Validation followed the applicable sections of the FDA and EMA recommendations. All experiments were performed with n = 6.

**Table S5:** EC_50_ [µM] and E_max_ values (± SD) of *CYP3A4* and *ABCB1* mRNA expression after exposure of LS180 cells to rifamycins for 24, 96, and 144 h.

|  | **Gene** | **Time point**  **[h]** | **EC_50_ ± SD**  **[µM]** | **E_max_ ± SD** |
| --- | --- | --- | --- | --- |
| **Rifampicin** | *CYP3A4* | 24 | 7.3 ± 0.44 | 3.5 ± 0.13 |
|  |  | **24*** | **32 ± 2.0** | **3.5 ± 0.13** |
|  |  | 96 | 3.2 ± 1.1 | 14 ± 1.3 |
|  |  | 144 | 4.8 ± 0.19 | 37 ± 2.8 |
|  | *ABCB1* | 24 | 8.6 ± 0.47 | 2.6 ± 0.068 |
|  |  | **24*** | **37 ± 2.0** | **2.6 ± 0.068** |
|  |  | 96 | 1.1 ± 0.056 | 5.1 ± 0.12 |
|  |  | 144 | 1.0 ± 0.065 | 7.0 ± 0.53 |
| **Rifabutin** | *CYP3A4* | 24 | 1.2 ± 0.24 | 2.1 ± 0.094 |
|  |  | **24*** | **Not fitted** | |
|  |  | 96 | 3.6 ± 0.84 | 6.3 ± 0.46 |
|  |  | 144 | 2.0 ± 0.28 | 7.5 ± 0.82 |
|  | *ABCB1* | 24 | 1.1 ± 0.027 | 1.4 ± 0.024 |
|  |  | **24*** | **Not fitted** | |
|  |  | 96 | 1.8 ± 1.1 | 2.8 ± 0.16 |
|  |  | 144 | 1.5 ± 0.72 | 4.5 ± 0.71 |

EC_50_: effective concentration, leading to 50 % of maximum effect [µM]; E_max_ (maximum effect) values are demonstrated as fold induction compared to untreated cells; **24*** = values generated by using corresponding intracellular concentrations of rifamycins. Data was fitted to an E_max_ model (four parameter-logistic equation with variable slope) using GraphPad Prism version 9.1.

**Table S6:** Concentration-dependent induction of CYP3A4 activity after exposure for up to 96 h in LS180 cells and CYP3A4 inhibition by rifamycins.

|  | **Time point**  **[h]** | **EC_50_ ± SD**  **[µM]** | **E_max_ ± SD** | **IC_50_ ± SD**  **[µM]** |
| --- | --- | --- | --- | --- |
| **Rifampicin** | 24 | 14 ± 4.2 | 3.5 ± 0.8 | 2.9 ± 0.85 |
|  | **24*** | **43 ± 1.5** | **3.5 ± 0.8** |  |
|  | 72 | 13 ± 3.1 | 7.9 ± 1.6 |  |
|  | 96 | 3.8 ± 0.58 | 4.1 ± 0.12 |  |
| **Rifabutin** | 24 | Not fitted | Not fitted | 11 ± 2.9 |
|  | **24*** |  |  |  |
|  | 72 |  |  |  |
|  | 96 |  |  |  |

EC_50_: effective concentration, leading to 50 % of maximum effect [µM]; E_max_ (maximum effect) values are demonstrated as fold induction compared to untreated cells; IC_50_: inhibitory concentration, leading to 50 % of inhibitory effect [µM]; **24*** = values generated by using corresponding intracellular concentrations of the rifamycins. Data was fitted to an Emax model (four parameter-logistic equation with variable slope) using GraphPad Prism version 9.1.

**Table S7:** Concentration-dependent activation (LS180 cells) and inhibition of Pgp by rifamycins (P388/dx cells).

|  | **Cell line** | **EC_50_/IC_50_ ± SD**  **[µM]** | **E_max_ ± SD** |
| --- | --- | --- | --- |
| **Rifampicin** | LS180 | 1.0 ± 0.0014 | 3.2 ± 0.37 |
|  | P388/dx | 13 ± 4.1 | 4.3 ± 0.53 |
| **Rifabutin** | LS180 | 1.1 ± 0.16 | 2.8 ± 0.48 |
|  | P388/dx | 0.32 ± 0.10 | 4.9 ± 1.3 |

EC_50_: effective concentration, leading to 50 % of maximum effect [µM]; E_max_ (maximum effect) values are demonstrated as fold induction compared to untreated cells; IC_50_: inhibitory concentration, leading to 50 % of inhibitory effect [µM]. Data was fitted to an E_max_ model (four parameter-logistic equation with variable slope) using GraphPad Prism version 9.1.
